# Notes and News

**Published:** 1889-03-30

**Authors:** 


### Notes and News.

Collected and Communicated.

The Hospital Annual will be ready next week.

The Index to Vol. V. of The Hospital will be issued next week with the first number of Vol. VI.

The new Cottage Hospital, which has been built at Epsom as a Jubilee memorial, will be opened on April 21st, by the Duchess of Teck. The designs are by Mr. J. R. Harding, C.E., and the building is neat and convenient, and should find plenty of supporters in a place like Epsom.

Mona's Isle has its cottage hospital and district nurse, "all of its own," as children say, and has just issued a satisfactory report of last year's work. Only one patient was received in the home, but the nurse paid 1,356 visits. Lady Gell is the founder and directress of this charity, and manages the finances so skilfully that though she never makes a public appeal, she has always a reserve to fall back upon.

A patient in the York County Hospital who was suffering from incurable heart disease, committed suicide last week by cutting his throat with a razor. Apparently he agreed with the Chinese, many of whom are killing themselves to avoid starvation, that anything was better than a lingering death.

The Hospitals Association is much to the fore at present. The scheme for supplying London with adequate ambulance organization is practical and useful, and has roused the public interest. £1,500 has already been subscribed for the plant and first year's expenses. Mr. H. L. Bischoffsheim has shown his appreciation of the good work done by the Association by presenting it with a donation of £250. This will help on some of the many excellent schemes which the Association is promulgating.

The fresco of Spring in the nurses' dining-hall at Guy's is nearly complete, and will soon gladden the eyes of the weary women who gather daily beneath it. A large picture from one of the wards is to be brought down to hang at the other end of the hall, and then the bare room will have a much more pleasant appearance. "Art in Hospitals" is a subject which might well be taken up by many men and women in want of something to do; for the Kyrle Society cannot effect a tithe of what is wanted in this way.

A very simple yet excellent charity is the Sea-Shell and Scrap-Book Missions, of 27, Benedict Road, Stockwell, and we recommend it to the attention of our younger readers. Christmas cards now out of season are wanted, and shells when the season comes. Through the generous help of many lady workers, these missions have now sent out 13,780 boxes and bags of shells and 22,044 scrap books. Card albums, framed cards, puzzles, envelope drawing books, and boxes of letters, etc., have also been distributed to the poor children of London and elsewhere.

St. Mary's Hospital at Manchester wants to celebrate its centenary by erecting a new building. Founded in 1790, this hospital has ever been one of the best training schools in midwifery and obstetric science, and here some of the first lectures to nurses were delivered by medical men. Twenty-two thousand pounds has been promised towards the new building, but there is some difficulty about the choice of a site. During the past year the patients admitted in the various departments were as follows:—In-patients, 764; home patients, 1,255; out-patients, 9,244; and maternity patients, 2,841; total, 14,104.

The committee of the Grosvenor Hospital are publishing a book, in connexion with the forthcoming "Al Fresco Fayre and Floral Fête," to be called the *Golden Grain Guide.* Sir Frederick Leighton, P.R.A., Mr. Harry Furniss, Miss Alma Tadema, Miss Clara Montalba, and others have kindly contributed original drawings for the book. Mr. Rider Haggard, Mrs. Kennard, the Countess of Munster, Lady Constance Howard, Violet Fane, John Strange Winter, Mr. Oscar Wilde, and other well-known literary people will write special stories or poetry. The book will be unique in shape and get up, and a first edition of 10,000 copies will be published.

Lady Dufferin last week paid a visit to the Royal Free Hospital, Gray's Inn Road, of which Lord Dufferin has been recently appointed president. Her ladyship is interested in the movement for the employment of women as doctors in India, and the authorities of the Royal Free Hospital allow the students of the Medical School for Women to use the hospital for instruction. The Royal Free should be kept before the public just now, because of the necessity for enlargement, and its want of funds. The "Wandering Minstrels" are going to give a concert at Holborn Town Hall on April 4th, in aid of the Royal Free, and all who desire to spend a pleasant evening, and benefit the hospital, should make a point of being present.

THE following vacancies are announced this week, the amount of salary in each case being given after the name of the institution:—

**Resident Medical Officers.**

Wallasey Dispensary. House Surgeon.—£120.
Western General Dispensary, Marylebone. Junior House Surgeon.—£75, lodging, etc.
Royal Southern Hospital, Liverpool. Junior House Surgeon.—£60, board, etc.
Newport Infirmary. House Surgeon.—£100, board, etc.
Liverpool Infirmary for Children. Assistant House Surgeon.—Board, etc.
Fisherton Asylum. Assistant Medical Officer.—£100, board, etc.
Scarborough Friendly Society. Medical Officer (married).—£200.
Birmingham General Hospital. Assistant House Surgeon.—Board, etc.
Salford Royal Hospital. Junior House Surgeon.—£50, board, etc.
City of London Hospital for Diseases of Chest. House Physician.

**Non-resident Medical Officers.**

City Dispensary. Physician.
Queen Charlotte's Hospital. Physician to Out-patients.

IN a letter to the *Standard* on the use of opium, Sir William Moore contends that in the malarious districts of India opium is not a curse, but a blessing. He says that opium has long been noted as an antiperiodic, and there is no manner of doubt that it is prophylactic against ague and malarious febrile diseases generally. If it were not for opium, the mortality from malarious fevers in India would be very much greater than it is. It is also an inestimable boon to the very poor, and especially in times of scarcity and famine, for it enables persons to exist upon less food than they otherwise could live on. Neither is the consumer of opium mentally incapacitated, if he does not indulge the habit to excess. This is certainly the other side to the opium question, of which the English public know little or nothing.

DR. J. R. JEAFFRESON has a strong appreciation of the beautiful. At the late Court of Governors of Warneford Hospital he warmly urged the purchase of a strip of land covered with a beautiful avenue of oak trees, now left just side the hospital boundary. He said that what had been acquired was only what was absolutely necessary to meet the wants of the hospital; and this avenue would not only add to the beauty of the locality, but would be an admirable promenade for convalescent patients. He expressed a strong hope that the public would respond to this suggestion of the medical staff, and provide the necessary funds to also secure the land on which this beautiful avenue stands. It is a pity that hospitals are such expensive luxuries—it is seldom they can afford to admire the beautiful.

THERE seems to be some idea that Hastings Hospital affairs are falling too much into the hands of the clergy. Eight out of twenty members of the board are clergymen, and last week another was added to the number. For our part we hope they will bring their experience of public affairs to bear, and insist upon such an alteration in the domestic arrangements as to secure the courteous treatment of all visitors, and the fullest facilities for seeing the whole of the hospital at all reasonable hours. This is a public institution, not a private house, and how many hundreds of pounds have already been lost to the charity by the extraordinary treatment visitors from a distance and others have experienced it would be difficult to estimate. If half we hear is true, there ought to be a change in the resident management without delay. The report of the first complete year's work done by this hospital is a good one, the medical statistics being very gratifying. There is still a debt of £3,000 on the building fund, and more annual subscribers are needed.

THE new hospital and sanatorium recently erected at West Heath by the King's Norton Rural Sanitary Authority, was formally opened on March 19th. The hospital buildings are erected on a plot of land of about six acres, enclosed by a boundary wall. The land and boundary wall cost about £1,400, and the buildings and other expenses connected therewith will amount to an additional £5,200. The buildings, in addition to the administrative offices, will accommodate twenty-eight adult patients, or about fifty-six children. Provision has also been made for the reception of private patients. Mr. Hawkes, in declaring the hospital open, referred to the benefit it should be to all dwellers in the district.

THE Hon. Louis Wingfield in his "Wanderings of a Globe-Trotter" tells some curious stories af medical practice in China. The hot-water cure at Kusaru seems to consist in literally boiling the patient alive; if the patient survives the treatment, however, the cure seems certain. The cases treated in the receiving-room of a hospital are thus described: "A tooth was out, an abscess was lanced, in a trice. Graver cases were diagnosed and sent to bed in an adjoining ward. Chop-chop—quick, quick! A basin—sponges—bandages! It was all so swiftly done that the bewildered patients were speechless. No chloroform was used. It seemed unnecessary, and again I was forced to remark, as I had done already at Canton, how comparatively unsensitive to pain is the Celestial."

THE tendency of the day is shown by a proposition lately brought before the committee of the Coventry Hospital: "That the time has now arrived, considering the large sums subscribed by the working men yearly to the funds of the hospital, that further power should be given to the committee of the working men in the general management of the institution." We hear on every side of similar resolutions, and we think all ought to be proud that at this crisis in hospital affairs it is the working men of England who are coming to the rescue. The old notion that the public—that is, the mass of mankind—and hospital supporters are synonymous terms is gradually disappearing. After careful inquiry, we have reason to believe that not more than five per cent. of our population give to hospitals, and these five are found either amongst the very wealthy or amongst our working men; and it is on the contributions of the last that hospitals will mainly have to depend. The mass of mankind are shamefully ignorant of their indebtedness to hospitals, or else they would surely subscribe in their thousands those guineas, or five guineas, which would render the income of some of our struggling institutions at least adequate to do the work entrusted to them.

THE governors of Birmingham General Hospital report that the very large number of 47,698 patients were treated at the hospital during the year 1888, of whom 33,059 were free, without ticket or recommendation. The funds of the hospital, which at the beginning of the year showed a deficiency of £6,847, have closed with the still larger adverse balance of £7,942, and this notwithstanding an increased contribution from the Hospital Saturday collection, and the receipt of £2,500 from the Musical Festival. The main cause of this deficiency is to be found in the vastly increased work which the hospital performs compared with that which it undertook a few years ago. The committee gratefully acknowledge the generous and costly gift which one of their members (Mr. J. C. Holder) has presented to the hospital. This gift consists of two new sanitary annexes (of four storeys each) to two of the wings of the hospital, which have been erected and equipped at the sole expense of the donor. Double the number of patients have been cured as compared with last year, and the proportion of deaths is much smaller. Lord Aylesbury was unanimously elected president, and the Bishop of Worcester and Mr. John Jaffray, J.P., vice-presidents.

